# Portable, non-invasive video imaging of retinal blood flow dynamics

**DOI:** 10.1038/s41598-020-76407-5

**Published:** 2020-11-19

**Authors:** Kyoung-A Cho, Abhishek Rege, Yici Jing, Akash Chaurasia, Amit Guruprasad, Edmund Arthur, Delia Cabrera DeBuc

**Affiliations:** 1 Vasoptic Medical, Inc., Baltimore, MD USA; 2 Department of Ophthalmology, Bascom Palmer Eye Institute, University of Miami Miller School of Medicine, Miami, FL USA; 3 Department of Ophthalmology, Bascom Palmer Eye Institute, University of Miami Miller School of Medicine, 1638 N.W. 10th Avenue, Room 509, Miami, FL 33136 USA

**Keywords:** Biomarkers, Medical research, Optics and photonics

## Abstract

Retinal blood flow (RBF) information has the potential to offer insight into ophthalmic health and disease that is complementary to traditional anatomical biomarkers as well as to retinal perfusion information provided by fluorescence or optical coherence tomography angiography (OCT-A). The present study was performed to test the functional attributes and performance of the XyCAM RI, a non-invasive imager that obtains and assesses RBF information. The XyCAM RI was installed and used in two different settings to obtain video recordings of the blood flow in the optic nerve head region in eyes of healthy subjects. The mean blood flow velocity index (BFVi) in the optic disc and in each of multiple arterial and venous segments was obtained and shown to reveal a temporal waveform with a peak and trough that correlates with a cardiac cycle as revealed by a reference pulse oximeter (correlation between respective peak-to-peak distances was 0.977). The intra-session repeatability of the XyCAM RI was high with a coefficient of variation (CV) of 1.84 ± 1.13% across both sites. Artery-vein comparisons were made by estimating, in a pair of adjacent arterial and venous segments, various temporal waveform metrics such as pulsatility index, percent time in systole and diastole, and change in vascular blood volume over a cardiac cycle. All arterial metrics were shown to have significant differences with venous metrics (p < 0.001). The XyCAM RI, therefore, by obtaining repeatable blood flow measurements with high temporal resolution, permits the differential assessment of arterial and venous blood flow patterns in the retina that may facilitate research into disease pathophysiology and biomarker development for diagnostics.

## Introduction

Retinal imaging and ensuing analysis of retinal status has been the mainstay for the diagnostics of ophthalmic ailments and has recently also been considered for its potential role in diagnostics and understanding of non-ophthalmic pathophysiology. Examples include brain-related conditions like Alzheimer’s Disease^1,2^ and lesions^3^, as well as cardiovascular conditions like hypertension^4^ and stroke^5,6^. Such progress, whether nascent or well-understood, has been enabled by technological advances that have continuously made more information available from the back of the eye. Color fundus photographs reveal a variety of morphological features that have diagnostic value—for example, exudates, hemorrhages, and microaneurysms (among others) are indicative of diabetic retinopathy^7–9^ and cup-to-disc ratio can provide insights into glaucoma^10^. Use of contrast agents like fluorescein or indocyanine green (ICG) provide perfusion status of vessels in addition to improving vessel-to-background contrast in the retina^11–14^. Scanning laser ophthalmoscopy (SLO) made retinal images available at a high spatial resolution^15^ while optical coherence tomography (OCT) has made it possible to image the back of the eye in three dimensions with high depth resolution^16–18^. Similarly, ultra-wide field (UWF) imaging has made large fields of view available for clinical decision making for diseases with symptoms in the peripheral retina^19^. Structural–functional changes can be visualized at a high resolution through the use of adaptive optics (AO)^20,21^. Commercial and research systems often integrate multiple modalities to obtain greater insights from the retina^22,23^. In contrast, imaging retinal physiology and function has only had moderate success until recently. Instruments that measured retinal blood velocities using the laser doppler phenomenon (e.g., Canon Laser Doppler Flowmeter)^24^ and particle tracking (e.g., Retinal Function Imager—RFI 3000)^25,26^ have suffered from poor repeatability limiting their use in the clinic and research. Recently, OCT angiography (OCT-A) has overcome reliability challenges^27,28^ permitting the assessment of perfusion in vessels without the use of contrast agents^29^, and by extension, assess non-perfused regions and vessel dropouts^30,31^. However, the limited temporal resolution of OCT-A prevents it from truly obtaining blood flow dynamics. Also, although vessel density analysis by OCT-A is widely used to characterize the retinal microvasculature network and perfusion, the density is not a scale invariant metric (i.e., variations in vessel diameter can alter density), and consequently it fails to be a suitable tool for comparison across dissimilar retinal tissue networks^32^. OCT with integrated Doppler modalities have been used to obtain total retinal blood flow, which is aggregated over the whole field of view^33–35^. The fastest type of OCT, which uses a swept source, has recently been shown to interpret patterns in blood flow^36^, but challenges remain^37^. Fluorescence-mediated approaches have produced absolute measures of blood flow but procedural complexity limit their widespread use for clinical medicine^38,39^.

Reliable imaging of retinal blood flow has the potential to complement traditional biomarkers with important clues that may enable disease diagnostics in the early stage prior to the development of vivid anatomical symptoms. In the case of glaucoma, while the causality is unknown, reduced blood flow has been known to correlate with nerve damage^40,41^, especially for normal tension glaucoma which is not easily diagnosed by a single test. Diabetic retinopathy is also characterized by reduced blood flow, notably even during early (mild to moderate) non-proliferative stage^42^. Interestingly, a differential comparison of flow in arteries and veins may offer additional insights^43,44^. Longitudinal assessment of physiological status and blood flow patterns may offer greater insights into disease status such as stage and severity^45,46^.

Laser speckle contrast imaging (LSCI) is a noninvasive technique that has shown promise in producing repeatable retinal blood flow measurements^47,48^. LSCI relies on inferring the rate of motion of blood by assessing the blurring in imaged speckle patterns when a tissue is illuminated by coherent light^49,50^. A commercially available retinal imager that uses laser speckle flowgraphy, the LSFG-NAVI (Softcare Co. Ltd., Japan)^51^, has displayed reduced blood flow in glaucoma^52^ and diabetic retinopathy^53^. A distinct advantage of LSCI is its ability to enable temporal assessment of the vasculature. Since retinal blood flow fluctuates over the duration of a heart beat, monitoring the temporal dynamics of retinal vessels may reveal additional changes in ocular pathologies.

We report the performance characteristics of a new portable, noninvasive investigational device, the XyCAM RI (Vasoptic Medical, Inc., Baltimore, USA) that implements laser speckle contrast imaging (LSCI) to assess blood flow in the retina. The present work characterizes the ability of the XyCAM RI to reliably reveal retinal blood flow dynamics with high spatio-temporal resolution and discriminate between arterial and venous blood flow patterns in the retina. We also present results obtained by the portable XyCAM RI in two different clinical settings: a primary care facility and a community setting.

## Results

### Clinical imaging, demographics, and sample size

Out of ten adult human subjects that consented to the study at each imaging site, imaging using the XyCAM RI was completed in ten subjects in the primary care setting (Site 1) and eight subjects in the community setting (Site 2). Complete data sets were not obtained in the remaining two subjects of Site 2 because one of the subjects had severe media opacity limiting our ability to acquire either XyCAM RI images or fundus photographs of acceptable quality, while the other subject withdrew from the study following unforeseen changes to their availability. This resulted in a total of eight subjects with complete imaging at Site 2. Both eyes were imaged for all subjects, resulting in retinal blood flow data collected from a total of 36 eyes across both sites.

**Figure 1:**
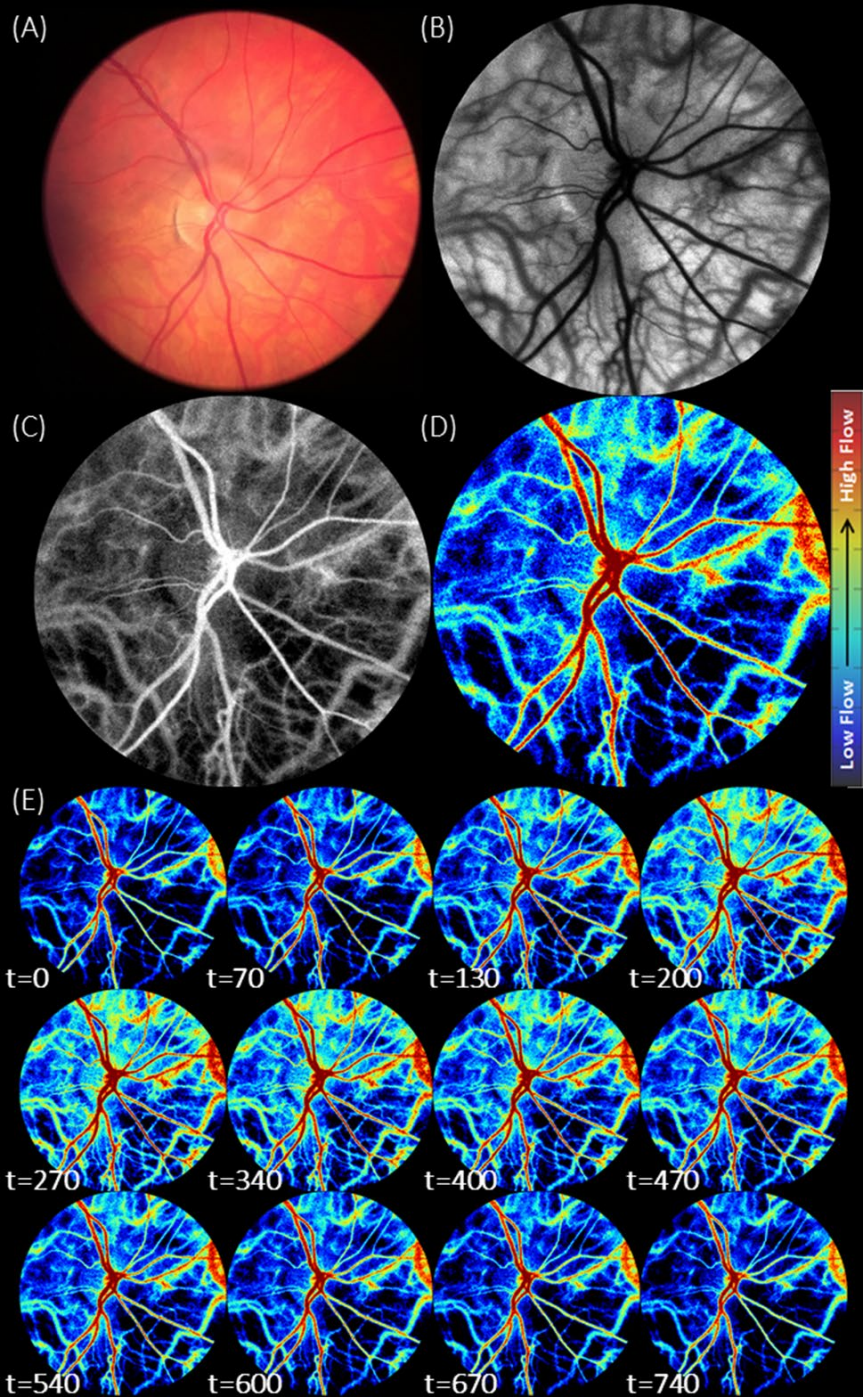
Images of the human retina, centered on the optic disc, obtained using multiple modalities. **(A)** Fundus photograph captured using a portable smartphone-based fundus camera model FOPNM-10 (Remidio Innovative Solutions); and **(B–E)** images obtained by the XyCAM RI (Vasoptic Medical, Inc.) using laser speckle contrast imaging to reveal blood flow. Specifically, **(B)** is a reconstructed fundus image revealing vascular morphology with high contrast and spatial resolution, **(C)** is a dye-free angiogram, and **(D)** reveals a blood flow velocity map (BFV) in the retina with **(E)** displaying blood flow changes across a full cardiac cycle with time in t = milliseconds.

Fundus photographs of each eye were also obtained for reference. Retinal image data was captured at 82 frames per second, and post-processed (not real-time) to reveal maps of blood flow velocity indices (BFVi), also available at a temporal resolution of 82 Hz. Figure 1 shows resulting images from an exemplary data set using a portable smartphone-based fundus camera (Fig. 1A) and a reconstructed fundus image (Fig. 1B), a dye-free angiogram (Fig. 1C), and a series of blood flow velocity images at time indices across a full cardiac cycle (Fig. 1D). Of the 18 adults, 16 were female and 2 were male, and the mean age across all subjects was 52.05 ± 17.75 years. The subjects had an average IOP measurement of 10.2 ± 3.5 mmHg, with a range of 6 to 19 mmHg. The average systolic blood pressure was 122.1 ± 19.8 mmHg, ranging from 100 to 181 mmHg, and the average diastolic blood pressure was 75.8 ± 11.6 mmHg, ranging from 60 to 107 mmHg. Two subjects had hypertension, one of which also had Type 2 diabetes. There were no subjects with heart diseases, and they had no significant ocular diseases. Although a history of hypertension or diabetes may affect blood flow, these subjects were included in the analysis, as the primary objective was to assess repeatability of the temporal dynamics of blood flow.

In 12 eyes, only the optic disc was analyzed, since no fundus images were recorded (4 eyes), or fundus image quality made arteries and veins indistinguishable (8 eyes). A total of 46 arterial ROIs and 46 venous ROIs outside of the optic disc ROIs were analyzed, eight of which were considered branched arteries and eight of which were considered branched veins. Ultimately, 3 out of 36 imaging sessions (8.3%) were omitted due to poor image quality, usually in the form of significant eye twitches. The correlation between age and diastolic, cycle mean, and systolic BFVi values in the optic disc was assessed and determined to be significantly negative (R = − 0.539, p < 0.01), significantly negative (R = − 0.373, p < 0.05), and inconclusive (R = − 0.214, p = 0.240) respectively and had inconclusive correlations with IOP (diastolic: R = − 0.203, p = 0.266; cycle mean: R = − 0.275, p = 0.127; systolic: R = − 0.312, p = 0.082).

### Repeatability of temporal measurements

Repeatability of temporal BFVi measurements was assessed by calculating the intra-session coefficient of variation (CV) of BFVi values across multiple cardiac cycles within a single imaging session. BFVi values were compared in consideration of their phase within the cardiac cycle, and therefore, separate assessments were done for systolic BFVi values, diastolic BFVi values, and BFVi values averaged over the cardiac cycle (“cycle mean BFVi values”). Region-specific intra-session CVs were computed to assess the repeatability of cycle mean BFVi values in the optic disc, arterial, and venous segments, which are reported in Table 1. The intra-session CV of cycle mean BFVi values within all assessed regions was 1.79 ± 0.93% in the primary care setting and 2.29 ± 1.61% in the community setting, while the combined intra-session CV was 1.84 ± 1.13%. Comparative CVs at systolic, cycle mean, and diastolic phases are reported in Table 2. A two-sample t-test revealed no significant differences between intra-session CVs at the two sites when considering diastolic (p = 0.593), cycle mean (p = 0.695), or systolic (p = 0.236) BFVi values.

**Table 1:** Intra-session CVs across different regions of interest for cycle mean BFVi values.

	Site 1 (n = 20 eyes) (%)	Site 2 (n = 16 eyes) (%)	Overall (%)
Artery	1.95 ± 0.89	2.07 ± 1.03	2.02 ± 0.95
Vein	1.32 ± 0.50	1.26 ± 0.75	1.29 ± 0.64
Branched artery	1.61 ± 0.45	2.10 ± 0.44	1.38 ± 0.74
Branched vein	2.15 ± 1.50	2.09 ± 0.22	1.84 ± 1.59
Optic disc	2.20 ± 1.49	2.28 ± 1.89	2.12 ± 1.52

**Table 2:** Intra-session CVs across different points of the cardiac cycle.

	Site 1 (n = 20 eyes) (%)	Site 2 (n = 16 eyes) (%)	Intra-CV (all sites) (%)
Diastole	2.73 ± 1.76	3.19 ± 2.23	2.63 ± 1.79
Cycle mean	1.79 ± 0.93	2.29 ± 1.61	1.84 ± 1.13
Systole	1.82 ± 1.10	2.36 ± 2.15	2.03 ± 1.47

### Analysis of blood flow trends over a cardiac cycle

Temporal patterns (“waveforms”) in BFVi measurements were compared with synchronously acquired pulse oximeter data by calculating the peak to peak (P–P) distances in each waveform. The correlation between the P–P distances of the pulse oximeter and the BFVi waveforms was 0.977 (p < 0.001) and the ratio of the P–P distance of the pulse oximeter to the BFVi waveforms was 0.99 ± 0.029. Heart rates of all subjects, as measured by the pulse oximeter exclusively, lay in the range from 52.90 to 98.62 beats per minute, and when estimated using BFVi waveforms exclusively, lay in the range from 52.34 to 99.54 beats per minute.

**Figure 2:**
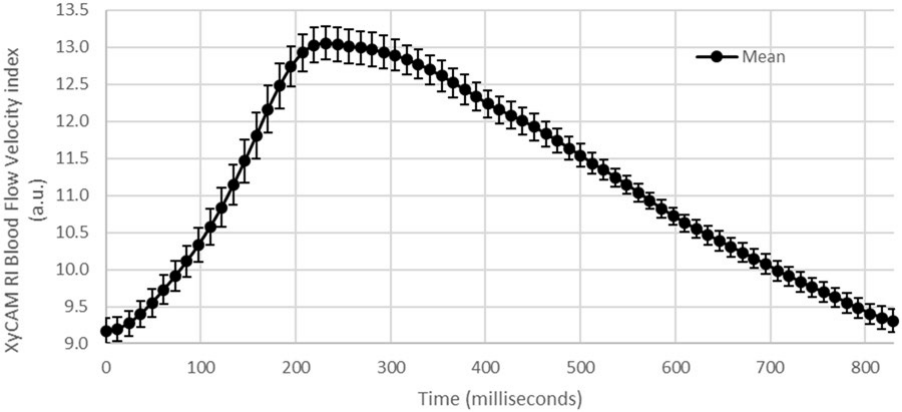
Temporal waveform of an optic disc region depicting amount of variation in the mean blood flow velocity index (BFVi) waveform across time with standard deviation.

For each isolated cardiac cycle within which mean BFVi values in the optic disc region did not have any obvious artifacts, the CV of each BFVi value was determined across all similar BFVi values in the same phase within each imaging session. Figure 2 displays the mean waveform at each point in time with the standard deviation highlighted for the different cardiac cycles detected within a single imaging session. In primary care clinic, the mean CV was 3.27 ± 1.74% and in community care setting, the mean CV was 3.12 ± 1.94%, and there was no significant different between the two sites (p = 0.837). Overall, across all the subjects, the mean CV was 3.19 ± 1.82%.

### Comparison of blood flow information in arteries and veins

Assessment of BFVi was carried out in regions of interest (ROIs) overlying arteries and veins emerging from the optic disc (“major” arteries and veins), as well as regions overlying arteries and veins past a branch point (“branched” arteries and veins). Specifically, pairs of adjacent arteries and veins were chosen that reasonably feed and drain from a similar region of the retina. For each ROI, the average diastolic, cycle mean, and systolic BFVi measurements were calculated and are displayed in Table 3. The cycle mean BFVi measurements across the disc, all arteries (major and branched), and all veins (major and branched) were calculated to be: 8.71 ± 1.68, 12.55 ± 2.53, and 13.03 ± 2.53 A.U. respectively. The BFVi measurements of major artery and vein pairs were compared using a paired student’s t-test, which revealed a significant difference between diastolic (p < 0.001) and cycle mean BFVi values (p < 0.05) but was inconclusive for systolic BFVi values (p = 0.160). There were no significant differences between branched artery and vein BFVi values.

**Table 3:** Systolic, diastolic, and mean BFVi values (A.U.) for major and branched arteries and veins and paired t-test between major artery and vein pairs and branched artery and vein pairs in systolic, diastolic, and cycle mean values.

	Major artery (n = 38)	Major vein (n = 38)	p-value	Branched artery (n = 8)	Branched vein (n = 8)	p-value
Diastole	10.02 ± 2.21	11.52 ± 1.89	p < 0.001	9.71 ± 2.79	10.54 ± 2.24	p = 0.252
Cycle mean	12.37 ± 2.49	13.23 ± 2.09	p < 0.01	11.97 ± 2.80	12.06 ± 2.56	p = 0.551
Systole	15.33 ± 3.09	14.95 ± 2.44	p = 0.160	14.24 ± 2.94	13.62 ± 2.98	p = 0.885

Additional waveform metrics were calculated for arteries and veins, including the normalized peak to trough BFVi value, percent time in systole and diastole, relative change in volume over a cardiac cycle, total relative blood flow volume over a cardiac cycle, and relative peak velocity. These metrics are displayed in Table 4 for major arteries and veins and in Table 5 for branched arteries and veins. For all metrics, the ratio of each artery to its adjacent vein is also shown in Tables 4 and 5. The metrics were tested using a paired student’s t-test to compare artery and vein pairs, and all metrics had significant differences between major arteries and veins (all p < 0.01) but only normalized peak to trough BFVi (p < 0.05), % time in systole (p < 0.05) and % time in diastole (p < 0.05) were significantly different between branched artery and vein pairs.

**Table 4:** Waveform metrics of major arteries and veins with artery to vein (A–V) ratios and results of a paired t-test.

	Major artery (n = 38)	Major vein (n = 38)	Major A–V ratio	p-value
Normalized peak to trough BFVi	0.42 ± 0.13	0.26 ± 0.08	1.64 ± 0.27	p < 0.001
%Time in systole	34.75 ± 4.98%	37.45 ± 4.16%	0.94 ± 0.14	p < 0.01
%Time in diastole	65.25 ± 4.98%	62.55 ± 4.16%	1.05 ± 0.89	p < 0.01
Relative change in volume (a.u.)	47.28 ± 18.28	42.23 ± 16.66	1.16 ± 1.16	p < 0.01
Total relative blood flow volume (a.u.)	241.14 ± 78.25	321.84 ± 92.67	0.77 ± 0.19	p < 0.001
Relative peak velocity (a.u.)	0.71 ± 0.23	0.54 ± 0.12	1.33 ± 0.34	p < 0.001

**Table 5:** Waveform metrics of branched arteries and veins with A–V ratios and results of a paired t-test.

	Branched artery (n = 8)	Branched vein (n = 8)	Branched A–V ratio	p-value
Normalized peak to trough BFVi	0.40 ± 0.16	0.25 ± 0.07	1.37 ± 0.47	p < 0.05
%Time in systole	33.34 ± 4.12%	36.81 ± 4.66%	0.80 ± 0.28	p < 0.05
%Time in diastole	66.66 ± 4.12%	63.19 ± 4.66%	0.96 ± 0.34	p < 0.05
Relative change in volume (a.u.)	38.85 ± 16.19	35.39 ± 21.56	1.14 ± 0.47	p = 0.801
Total relative blood flow volume (a.u.)	219.46 ± 114.26	263.24 ± 134.93	0.82 ± 0.33	p = 0.151
Relative peak velocity (a.u.)	0.73 ± 0.18	0.60 ± 0.10	1.02 ± 0.35	p = 0.185

The time delay between the temporal waveforms of arteries and veins was assessed at diastolic, cycle mean, and systolic timestamps and calculated across all artery and vein pairs. The average delay between major arteries and veins was calculated as 21.91 ± 13.83 ms at diastole, 50.65 ± 20.68 ms at cycle mean, and 47.99 ± 40.07 ms at systole (z-test: all p < 0.001) while the average delay between branched arteries and veins was calculated as 19.60 ± 17.50 ms at diastole, and 54.59 ± 24.84 ms at cycle mean, and 57.20 ± 39.16 ms at systole (z-test: all p < 0.005).

## Discussion

The XyCAM RI is able to obtain images of retinal blood flow at a high temporal resolution permitting the analysis of blood flow fluctuations associated with a cardiac cycle. This information is complementary to the perfusion metrics such as perfusion density or vessel length density obtained by OCT-A. The BFVi measurement obtained by the XyCAM RI represents blood velocity integrated along the depth dimension, but not along the width of the vessel. Therefore, blood flow estimates for any specific vessel segment are obtainable by integrating the BFVi values along the transverse diameter of the vessel^54^. Similarly, mean velocity through the vessel can be computed by dividing the estimated blood flow by the cross-sectional area of the vessel. However, as a middle ground between velocity and flow, the BFVi measurement provides a good estimate of the perfusion dynamics where both velocity and vessel caliber are important. The high correlation of P–P distances in the temporal trends of BFVi and the plethysmogram validates that the BFVi oscillations are associated with the increase and decrease of flow rates within a cardiac cycle. The ability to discriminate between systolic, cycle mean, and diastolic flow may add additional information regarding capillary filling than temporally averaged perfusion information available with OCT-A. The phase lag between the BFVi waveform and the plethysmogram arise from the different path lengths of the eye and finger from the heart. XyCAM RI is able to detect pulsatile changes with a high temporal resolution. Although the waveforms cannot be compared, the delay between the peaks may be caused by differences in heart rate as well as height, and the overall range of distances display the large variation of heart rates both devices are able to acquire.

While every pixel of the BFVi images (Fig. 1e) represents a square region of side 6.27 ± 0.43 µm on the retina, speckle contrast calculation aggregates information from 5 × 5 pixels, that is, from a 31.35 µm × 31.35 µm region on the retina. Therefore, the XyCAM RI does not resolve vessels with diameters less than ~ 33 µm. However, information pertaining to flow velocities in such unresolved low caliber vessels is not lost, and instead assessable in the form of mean BFVi in custom-selected regions. Indeed, such regional mean BFVi values also exhibit fluctuations that are synchronized with the cardiac cycle.

During the course of the reported study, the XyCAM RI was transported and installed multiple times at the imaging sites on general purpose tables without any provisions for height adjustment or any additional stabilizing features. Regardless, intra-session repeatability was high at both imaging sites, and notably, the observed CV of 2.43 ± 2.03% in BFVi measurements from the optic disc is on par or better than the previously reported CV of 3.4 ± 2.0% in blood flow index measurements using the LSFG-NAVI (Softcare Co. Ltd., Japan), another ophthalmic imaging instrument that uses a speckle-based approach^48^. Both the LSFG-NAVI and the XyCAM RI produce relative measurements of blood flow, unlike some other technologies^25,55–57^ that claim to provide absolute measurements of blood flow or velocities measurements. However, these absolute measurements are obtained with lower repeatability, such as the Canon Laser Blood Flowmeter (CV of 16.4 ± 12.8%)^55^, the Retinal Function Imager (RFI) 3000 (mean CV of 10.9%^25^ and 22.5%^58^), and a Laser Doppler Velocimetry measurements (mean CV of 18%)^56^ which has limited their use in clinics. Although LSCI produces relative measurements of blood flow, the temporal dynamics of vascular flow revealed the XyCAM RI are highly repeatable, as shown in this study. A previous study has been conducted to compare the validatity of XyCAM RI blood flow measurements against the RFI, which resulted in a correlation of r = 0.80 in an in-vitro blood flow setup, but the study was limited in comparison of blood flow metrics in clinical data due to high variability in RFI measurements^26^. The repeatability results also showed improvements in the XyCAM RI design from previous studies with a pilot device that displayed a mean intra-session CV of 8.71 ± 2.55% within the overall vascular component using a stabilized system and 10.41 ± 3.50% in a handheld system^59^. Although the present XyCAM RI system is not designed to be used in a handheld configuration, the system is portable and easily stabilized on a benchtop, for effective use in a primary care clinic, a community care center, or in a shared environment.

Differential assessment of arterial and venous blood flow may provide insights into retinal circulation and vascular status that are useful in disease diagnostics^42,44,60^. The ability to capture blood flow dynamics over a cardiac cycle enables the XyCAM RI to produce additional metrics based on rising and falling trends in blood flow that could be investigated for their potential role as biomarkers. Higher standard deviations observed in volumetric and peak velocity measurements reported in Tables 4 and 5 may be attributed to physiological fluctuations rather than the inherent repeatability of the technology. In particular, because volumetric measurements integrate BFVi values over time, variation is expected to be higher. Although major arteries and veins displayed significant differences in measurements displayed in Table 4, branched arteries and veins did not display significant differences in relative volume change, total relative blood flow volume, and relative peak velocity, as seen in Table 5. This finding may be attributed to physiological differences between larger caliber arteries and veins being more significant than smaller caliber arterioles and venules, but could also be the effect of the smaller sample size of paired branches (n = 8) than major vessel pairs (n = 38). Additionally, these metrics, not only in the artery and veins but also in regions such as the optic disc, may reveal early evidence of disease, as reported in the case of patients with normal tension glaucoma^61^.

The purpose of this study was to primarily assess and report the temporal characteristics of retinal blood flow data obtained by the XyCAM RI, and therefore, comparisons have been made intra-session. Further studies will need to be conducted to assess the XyCAM RI’s repeatability and reproducibility across multiple sessions as well as in longitudinal studies. Another limitation of this study is the relatively small and controlled study population. While data from eighteen subjects have provided us with statistically significant trends, additional studies are necessary to evaluate performance in eyes with varying degrees of pigmentation and pathology in individuals with greater variations in age and heart rates. Additionally, the XyCAM RI produces relative values, as opposed to true velocity and flow measurements, that may also be influenced by characteristics of the eye, such as refractive error and pupil size, therefore, inter-patient comparisons may require calibration^51,62^. The XyCAM RI is designed to image the retina through a pupil with diameter greater than 4 mm. Additionally, depending on the characteristics of the retinal pigment epithelium, the XyCAM RI may also reveal flow information in choroidal vessels, especially those portions of choroidal vessels that do not underly major retinal vessels. The XyCAM RI is also susceptible to motion artifact and fast eye twitches are particularly problematic because they cannot truly be compensated for. Therefore, its use may be restricted in individuals with nystagmus or other conditions where subjects may have difficulty fixating their gaze for the duration of at least one cardiac cycle. In our study, 3 out of 36 imaging sessions (8.3%) were omitted due to poor image quality resulting from eye twitches.

Overall, our study demonstrated the ability of the XyCAM RI to obtain reliable and repeatable information pertaining to blood flow dynamics in the retina that may complement currently available information and lead to novel insights.

## Methods

### Human subjects

All clinical investigations were carried out in accordance with protocols approved by the University of Miami Institutional Review Board (IRB). Only those subjects that provided informed consent were enrolled into the study and underwent retinal imaging sessions. The study was conducted at two different sites: Site 1—Primary Care Physician’s office (primary care facility); and Site 2—Easter Seals Adult Care Center (community setting). Ten healthy subjects were recruited and enrolled at each of the two sites following informed consent.

At each of the two sites, the XyCAM RI was used to acquire data from two circular retinal fields – one centered on the optic nerve head (ONH) and one centered on the fovea – from each eye of each subject. Mydriatic drops (1% Tropicamide Solution) were administered to dilate all subjects. For reference, a fundus image was captured using a portable smartphone-based fundus camera model FOPNM-10 (Remidio Innovative Solutions)^63,64^ as typically used in these settings (Fig. 1A).

### Image acquisition procedure

For each imaging session, the patient rested his/her head on a standard ophthalmic chin rest and the XyCAM RI was maneuvered to focus on the desired retinal fields. The patient was instructed to fixate on an external target (LED attached to the XyCAM RI) for imaging retinal fields centered on the ONH and on an internal target (blinking X inside the XyCAM RI) for imaging retinal fields centered on the fovea. Upon achieving satisfactory focus, the patient was instructed to remain fixated at the target and try to refrain from blinking or any eye movements, and an imaging session was initiated. Image data was continuously acquired over a duration of six seconds at a frame rate of 82 frames/second. Because the XyCAM RI illuminates the patient’s retina with non-scanning, continuous-wave laser light (peak wavelength in the range: 780–790 nm, laser power in the range: 1.4–1.6 mW) during image acquisition, obtained image data captures speckle patterns. In regions with blood motion (flow), a blurring effect is observed in the speckle pattern that can be computationally processed to obtain estimates of the rate of blood motion (flow)^50,65^. For reference, a finger plethysmogram including heart rate and blood oxygen saturation data was simultaneously and synchronously recorded using a finger pulse oximeter, an optional accessory to the XyCAM RI. The data was rapidly processed and presented to the operator for quality assessment so that the operator could determine whether to accept the data as recorded or repeat the image acquisition session. Artifacts caused by patient eye movement or blinking are often evident during this rapid review.

### Image analysis procedure

Extent of blurring in the speckle pattern at any location (pixel) and time (frame) is estimated by computing *speckle contrast* at the specific pixel in a specific frame, defined as the coefficient of variation of pixel intensities within the pixel’s spatio-temporal neighborhood (in the image frame, and in temporally adjacent image frames). Thus, each raw laser speckle data stack of six second duration acquired by the XyCAM RI, was processed using multiple speckle contrast calculation algorithms, which have been described in detail previously^65^. A reconstructed fundus image (Fig. 1B) is obtained by processing for speckle contrast at each pixel in the image across 82 speckle image frames and displays high spatial resolution and distinct morphological features. A dye-free angiogram (Fig. 1C) is a visualization comparable to traditional dye-based angiography, and commensurately depicts regions of high and low flow in grayscale. Lastly, a colored “Blood Flow Velocity” (BFV) image stack (Fig. 1C) is produced by calculating speckle contrast at each pixel using pixel intensities in a 5 pixel × 5 pixel window around it in a sliding set of 15 frames, and mapping speckle contrast values to BFV values as described previously^47^, to display changes in blood flow across multiple cardiac cycles. The BFV image displays blood flow velocity indices and is used for all following data analyses.

For each of the processed data sets, images were registered to a user defined frame such that motion across time was minimized using the XyCAM RI Software. This registration algorithm is able to detect and correct for slow motion such as motion caused by the drifting of focus, but not rapid motion that manifests in the number of frames needed to generate a single BFV image. For saccades and quick eye twitches, vascular information is lost, and therefore assessment is carried out in those portions of the six second data that are not affected by sudden motion.

**Figure 3:**
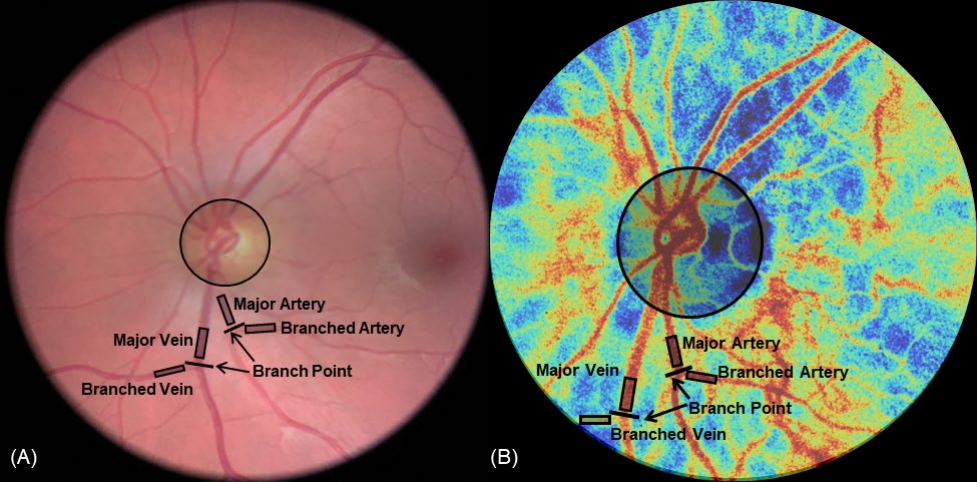
Description of assessed regions of interest. Regions selected and labeled as optic disc, major arteries and veins, and branched arteries and veins in: **(A)** fundus photograph obtained using portable smartphone-based fundus camera model FOPNM-10 (Remidio Innovative Solutions); and **(B)** retinal BFV image obtained by the XyCAM RI (Vasoptic Medical Inc.) (contrast altered for emphasis of selected regions).

Multiple regions of interest (ROIs) were manually selected around the ONH: one elliptical region around the optic disc and rectangular regions overlying at least one artery and vein pair immediately outside the optic disc region prior to bifurcation. If within the field of view, an artery and/or vein was also selected immediately following the first bifurcation. The lengths of the rectangles were standardized while the widths were defined by the width of the vessel of interest. An example of selected regions of major and branched vessels are displayed in Fig. 3. When examing vessels in the retina, these vessels may be arterioles or venules, based on vessel size and level of branching. However, all vessels are referenced as arteries and veins for consistency. After selection, the mean within each ROI was calculated for each BFV image frame in the six second dataset to obtain a time dependent blood flow velocity index (BFVi—arbitrary units) value that fluctuates with the cardiac pulse (Fig. 2). This waveform, combined with the BFV images in a video format, were examined to define acceptable data subsets each depicting a single cardiac cycle from a trough to the next trough in BFVi values. The cardiac cycles were initially selected based on automated recommendations from the XyCAM RI software. The endpoints (two adjacent troughs) of each cardiac cycle were refined manually if needed, and some cardiac cycles that contained visibly obvious artifacts from eye motion, corneal glare, or blinking, were excluded from analysis.

Across all the ROIs, an average and standard deviation of the diastolic (trough), cycle mean, and systolic (peak) values were calculated across all acceptable data subsets (cardiac cycles) for a given session. The intra-session repeatability was calculated using the coefficient of variation (CV) of each diastolic, cycle mean, and systolic BFVi value. The CV was calculated as the standard deviation of a given set of BFVi values divided by the mean of the set of values. The average CV was assessed for each region type and across the two sites using the cycle mean BFVi value.

**Figure 4:**
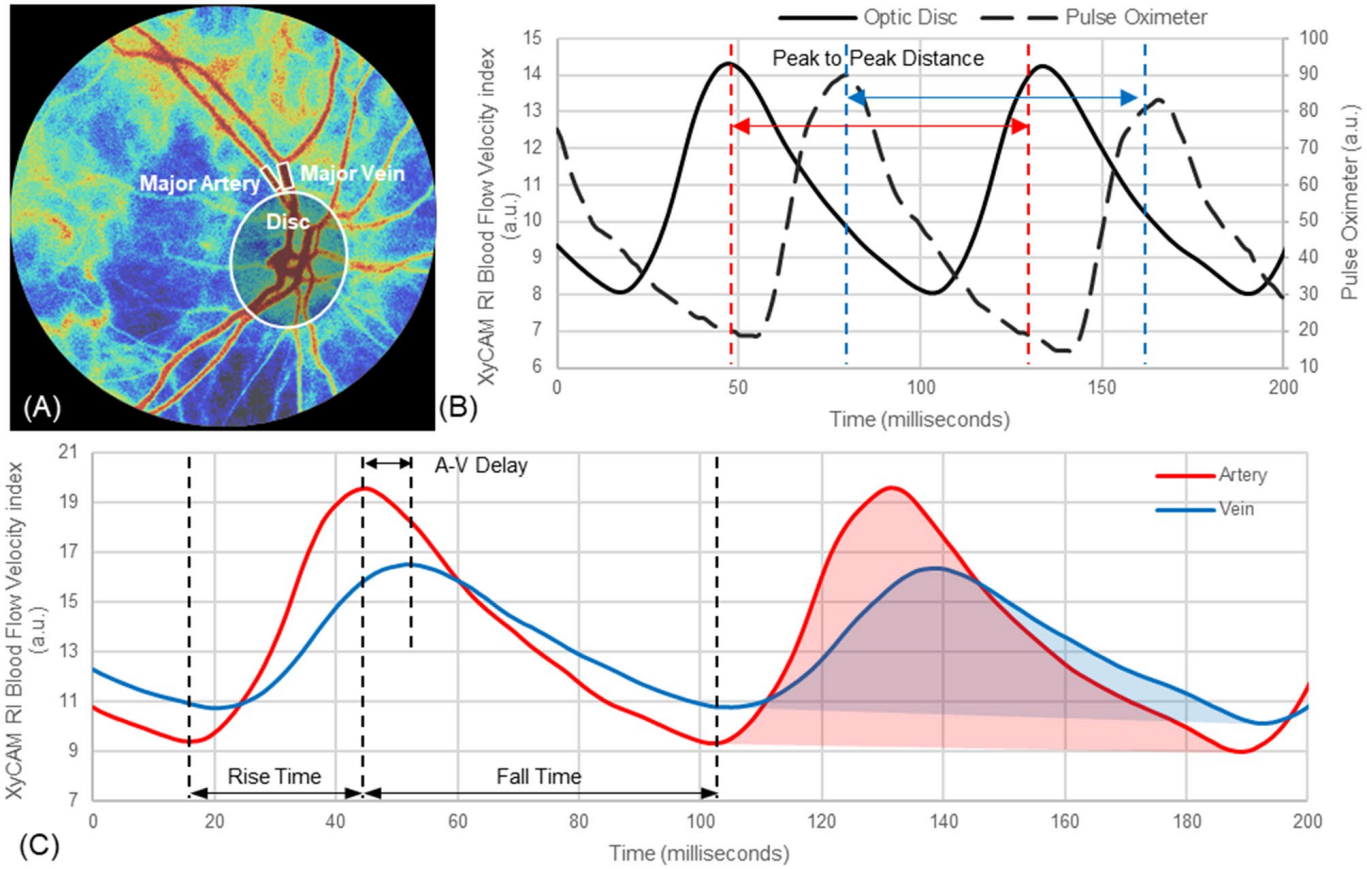
Differential assessment of flow dynamics in retinal arteries and veins. **(A)** Retinal BFV image shows assessed regions, namely: optic disc (Disc), a significant artery and vein pair (Major Artery and Major Vein). **(B)** Temporal trends (waveforms) of BFVi aggregated over the Disc region and the plethysmogram captured by a reference Pulse Oximeter reveal a lag, measured by peak to peak distance. **(C)** BFVi waveforms in a major artery and vein pair reveal the artery-to-vein transit delay between the peak flow rates. Additionally, times for the flow rate to peak (rise time) and fall to its minimum (fall time) are also determined to characterize flow dynamics. The shaded regions represent the change in blood volume in the artery and vein respectively during a cardiac cycle.

Waveforms obtained by aggregating BFVi values in an ROI consisting of the optic disc were compared with temporal data obtained synchronously from a finger pulse oximeter (CMS60C, Contec Medical Systems). The peak-to-peak (P–P) distance between cardiac cycles were measured for both modalities and the ratio of the P–P distance of the pulse oximeter to the BFVi waveforms was tested (Fig. 4B) for possible correlation. For each of such BFVi waveforms aggregated over the optic disc, BFVi values were compared with other BFVi values in the same phase of each acceptable cardiac cycle within the imaging session to characterize a secondary CV that captures the fidelity of the temporal resolution.

Adjacently located arteries and veins that feed and drain approximately the same region of the retina were examined in pairs in order to see if there were any significant differences in retinal blood flow fluctuations between the two types of vessels and the temporal sensitivity and limitations of the XyCAM RI. The time delay between BFVi peaks in arterial ROIs and venous ROIs was analyzed based on the output waveform metrics. The following waveform metrics were compared across arteries and veins: the percent of rise and fall time, the amount of peak to trough fluctuations, the relative change in volume fluctuation, the relative total blood flow volume, and the relative peak velocity values. All metrics obtained from arterial ROIs and venous ROIs were pairwise tested for statistical differences using a paired student t-test. A p-value < 0.05 was considered significant. Figure 4 displays the regions assessed and metrics used for artery and vein comparisons.
